# Deep learning and feature based medication classifications from EEG in a large clinical data set

**DOI:** 10.1038/s41598-020-70569-y

**Published:** 2020-08-26

**Authors:** David O. Nahmias, Eugene F. Civillico, Kimberly L. Kontson

**Affiliations:** 1 Electrical and Computer Engineering, University of Maryland, College Park, MD 20740 USA; 2 Center for Devices and Radiological Health, US Food and Drug Administration, Silver Spring, MD 20993 USA; 3 National Institutes of Health, Bethesda, MD 20892 USA

**Keywords:** Machine learning, Computational neuroscience

## Abstract

The amount of freely available human phenotypic data is increasing daily, and yet little is known about the types of inferences or identifying characteristics that could reasonably be drawn from that data using new statistical methods. One data type of particular interest is electroencephalographical (EEG) data, collected noninvasively from humans in various behavioral contexts. The Temple University EEG corpus associates thousands of hours of de-identified EEG records with contemporaneous physician reports that include metadata that might be expected to show a measurable correlation with characteristics of the recorded signal. Given that machine learning methods applied to neurological signals are being used in emerging diagnostic applications, we leveraged this data source to test the confidence with which algorithms could predict, using a patient’s EEG record(s) as input, which medications were noted on the matching physician report. We comparatively assessed deep learning and feature-based approaches on their ability to distinguish between the assumed presence of Dilantin (phenytoin), Keppra (levetiracetam), or neither. Our methods could successfully distinguish between patients taking either anticonvulsant and those taking no medications; as well as between the two anticonvulsants. Further, we found different approaches to be most effective for different groups of classifications.

## Introduction

Machine learning applied to medical research and treatment has the potential to revolutionize the patient-care continuum. From diagnosis to prognosis, treatment to rehabilitation, advanced algorithms are enabling more patient-specific care by leveraging existing data from patients^1^. With this emerging analytical approach nipping at the heels of medical practice, more portable, compact medical devices are being developed to capture physiological and/or movement data from individuals within controlled laboratory settings or in the real-world that can be used as input in machine learning algorithms^2^.

Electroencephalography (EEG) is one example of an application space with a high potential for impact from machine learning, as this device technology is increasingly being used for diagnostic and rehabilitative purposes^3,4^, and as a surrogate biomarker for pharmacodynamic modelling of drugs^5–7^. EEG is typically non-invasive, relatively inexpensive, and allows for direct tracking of neural population activity with high temporal resolution for objective measurements of cognitive function and communication between brain regions^8^. Although research into applications of machine learning to EEG has been underway for a decade, more work is needed to understand its limits in deriving information that could inform clinical decisions. Specifically, adequate validation data sets with known subject or patient characteristics are necessary in order to assess the accuracy and adequacy of machine learning approaches to clinical problems. Up to the present, this necessity has limited the size of most research studies to a small number of patients. Fortunately, the creation of large clinical databases with both patient information and corresponding physiological data will now enable deeper exploration of many classification problems with clinical relevance. One such database is the Temple University Hospital (TUH) EEG Corpus.

The TUH corpus is an unprecedented data set composed of thousands of subject recordings collected under conditions less constrained than those typically employed in laboratory research settings^9^. It primarily consists of data from subjects with EEG abnormalities, with many subjects taking anticonvulsant medications. Recent studies have used the TUH corpus data set to develop models to automatically analyze EEG^10^, classify EEG pathology status^11^, and detect different seizure types and events using both feature-based^12^ and deep learning-based^13^ methods. All this provides the means to explore novel medication state classifications by evaluating the neurophysiological signals of thousands of subjects. The ability to discern medications taken by an individual through neurophysiological features alone could have benefits in emergency medicine (e.g. determining appropriate life-saving interventions), obtaining more objective and accurate medication reporting, and predicting treatment outcome and effectiveness^14^. Can EEG alone be used to differentiate someone taking a certain medication from someone not taking any medication? Can EEG alone be used to differentiate individuals taking two different medications from the same drug class (e.g. anticonvulsants, opioids)? More generally, what types of biomedical questions are amenable to conclusive retrospective analysis of EEG data? With the increasing availability of large sets of clinical EEG data, we can begin to explore these questions.

When applying machine learning, there are generally two different algorithmic approaches to consider: deep learning or feature-based algorithms, each with advantages and disadvantages. Since EEG data can be difficult to interpret, even by experts, the automated feature extraction provided by deep learning network based models such as a simple linear neural networks (LNN), a shallow convolutional neural networks (SCNN), a deep convolution neural networks (DCNN), and EEGNet may be advantageous and require less knowledge about the data set and signal to perform well^15^. When expert knowledge about the data and signals are available, application-specific features can be crafted and used in a feature-based approach such as support vector machines (SVM), kernel SVM (kSVM), and random forests (RF). Feature-based methods have been shown to perform well in EEG applications and to produce consistent results^16,17^. Thus, to determine potential differences in classification performance and impact on understanding the underlying clinical implications, both feature-based and deep learning models were investigated and compared as previously done in novel classification paradigms^18–20^.

The goals of this work were to (1) determine if machine learning can predict medication states from neurophysiological activity captured through EEG and (2) compare the accuracy of feature-based and deep learning classification methods. By investigating the abilities of different machine learning algorithms to differentiate individuals taking different medications though neurophysiological signals provided through EEG, this work is a step on the road to the broader utility of machine learning in biomarker development.

## Results

We report our classification results in Table 1 from feature-based and deep learning neural network classifiers on different medications for both normal EEG and abnormal EEG populations. We report the mean and standard deviation of the 10-fold cross validation test accuracy results as a way to represent how the method best generalized on unseen data. We further report the P-value from Kruskal–Wallis tests between the test results obtained from correctly labeled training data and randomly labeled training data.

**Table 1:** Mean ± standard deviation percent test classification accuracies and significance (P-value) for six different classifications.

Classification	*n*-total (n-test)	Feature-based methods			Network-based methods			
		SVM % (*P*-value)	kSVM % (*P*-value)	RF % (*P*-value)	LNN % (*P*-value)	SCNN % (*P*-value)	DCNN % (*P*-value)	EEGNet % (*P*-value)
Dilantin vs. Keppra with Normal EEG	350 (34)	$51.18\pm 6.47$ ($P<.939$)	$58.23 \pm 5.23$ $*$ ($P<.004$)	$\mathbf {58.53} \pm \mathbf {4.25}$ ($P<.013$)	$51.47 \pm 7.70$ ($P<.699$)	$53.24 \pm 7.94$ ($P<.117$)	$55.59 \pm 8.77$ ($P<.466$)	$49.12 \pm 8.63$ ($P<.074$)
Dilantin vs. Keppra with Abnormal EEG	528 (52)	$57.50\pm 3.69$ $*$ ($P<.009$)	$57.88 \pm 5.99$ $*$ ($P<.009$)	$\mathbf {64.23} \pm \mathbf {6.58}$ $*$ ($P<.006$)	$50.77 \pm 6.09$ ($P<.704$)	$56.35 \pm 6.02$ $*$ ($P<.003$)	$60.19 \pm 4.94$ ($P<.046$)	$39.42 \pm 8.21$ ($P<.062$)
Dilantin vs. No medications with Normal EEG	358 (34)	$$58.61\pm 5.75$$ $$*$$ ($$P<.004$$)	$$59.17 \pm 6.34$$ ($$P<.011$$)	$$61.94 \pm 6.58$$ $$*$$ ($$P<.006$$)	$$47.94 \pm 8.43$$ ($$P<.877$$)	$$60.00 \pm 7.69$$ $$*$$ ($$P<.008$$)	$$\mathbf {66.76} \pm \mathbf {6.58}$$ $$**$$ ($$P<.001$$)	$$54.12 \pm 5.61$$ ($$P<.378$$)
Dilantin vs. No medications with Abnormal EEG	640 (64)	$$66.72\pm 6.78$$ $$**$$ ($$P<.001$$)	$$\mathbf {70.78} \pm \mathbf {3.28}$$ $$**$$ ($$P<.001$$)	$$70.00 \pm 2.95$$ $$**$$ ($$P<.001$$)	$$52.66 \pm 3.05$$ ($$P<.638$$)	$$64.53 \pm 5.68$$ $$**$$ ($$P<.001$$)	$$68.59 \pm 6.11$$ $$**$$ ($$P<.001$$)	$$45.00 \pm 3.81$$ ($$P<.493$$)
Keppra vs. No medications with Normal EEG	350 (34)	$$55.59\pm 4.45$$ ($$P<.023$$)	$$56.47 \pm 8.50$$ ($$P<.120$$)	$$57.65 \pm 7.69$$ ($$P<.339$$)	$$51.18 \pm 6.20$$ ($$P<.702$$)	$$55.88 \pm 6.44$$ ($$P<.065$$)	$$\mathbf {62.94} \pm \mathbf {6.34}$$ $$**$$ ($$P<.001$$)	$$49.12 \pm 8.22$$ ($$P<.646$$)
Keppra vs. No medications with Abnormal EEG	528 (52)	$$71.54\pm 5.76$$ $$**$$ ($$P<.001$$)	$$\mathbf {73.46} \pm \mathbf {3.92}$$ $$**$$ ($$P<.001$$)	$$70.00 \pm 4.80$$ $$**$$ ($$P<.001$$)	$$48.84 \pm 5.17$$ ($$P<.820$$)	$$70.77 \pm 3.42$$ $$**$$ ($$P<.001$$)	$$68.65 \pm 6.66$$ $$**$$ ($$P<.001$$)	$$51.92 \pm 7.60$$ ($$P<.704$$)

*n*-total represents all subjects used in training and testing while *n*-test were number of subjects used exclusively for testing. Kruskal–Wallis *P*-value significance thresholds set at: *$P < .01$, **$P < .001$. Bold values are the highest mean accuracies for each classification.

**Figure 1:**
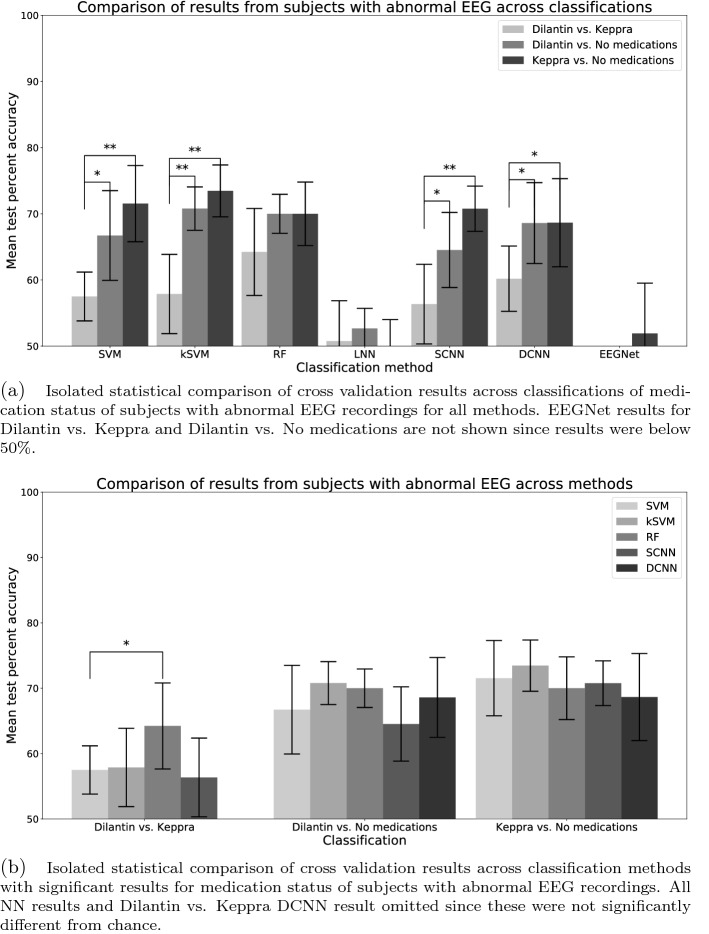
Isolated statistical comparisons of results for subjects with abnormal EEG recordings: (**a**) across classifications, and (**b**) across classification methods with significant results. Kruskal–Wallis test *P*-value significance thresholds set at: *$P<.01$, **$P< .001$. Unmarked comparisons within groups were non-significant.

Results show that different models yielded the highest levels of accuracies for different classification groups. Except for the simple LNN and EEGNet (column 6 in Table 1), classification accuracy results for all other methods were found to be statistically significant from chance for the majority of classifications explored. The comparison of classification accuracies across the seven methods and across a given classification task, however, had more variable results. When comparing individuals taking Dilantin versus individuals taking Keppra (rows 1 & 2 in Table 1) kSVM and RF models found significant classifications, with RF models obtaining the highest levels of classification. Comparing individuals taking Dilantin versus individuals taking no medications or individuals taking Keppra versus individuals taking no medications, the classification accuracy and significance of the result generally increased. We found that DCNN models obtained the highest levels of accuracies for classifications comparing subjects with normal EEGs taking a single medication versus those taking no medications. As expected, the loss of these models decreased quickly over the first few epochs after which changes were small until the stopping criteria were met. On the other hand, kSVM models yielded the highest levels of accuracies when comparing subjects with abnormal EEGs taking a single medication versus those taking no medications.

From these results we see that the classification accuracies of models for subjects with abnormal EEG were generally higher (rows 3, 5, and 7 in Table 1) than the analogous classifications for subjects with normal EEGs (rows 2, 4, and 6 in Table 1). To better assess discriminability of these classifications from the abnormal EEG data set, an isolated statistical test was performed using the vectors of classification accuracies from the 10-fold cross validations. Figure 1a shows the results of Kruskal–Wallis tests between these three classifications for all models investigated. The results of this additional statistical test showed that the two anticonvulsants had the same levels of discriminability since no significant differences were found between subjects taking Dilantin versus subjects taking no medications and subjects taking Keppra versus subjects taking no medications. However, the accuracies from abnormal EEG recordings of subjects taking Dilantin versus subjects taking Keppra were significantly different from both accuracies of subjects taking Dilantin versus subjects taking no medications and subjects taking Keppra versus subjects taking no medications for all methods except RF, LNN, and EEGNet models ($P < .01$) (Fig. 1a).

Comparing the accuracies of each classification with abnormal EEG between methods, several methods were found to be significantly different from other methods. Figure 1b shows the results of Kruskal–Wallis tests between the 10-fold cross validations of methods with significant results for each classification. We omit LNN and EEGNet from these analyses since as mentioned they did not find any significant results and see that only RF were significantly different than SVM results for comparing subjects with abnormal EEGs taking Dilantin versus those taking Keppra. We also note that for significant results the standard deviation of the feature-based results were, on average, 0.96% smaller than those from the corresponding deep learning models.

For the two classifications where kSVM had the highest accuracies, comparing subjects with abnormal EEGs taking a either Dilantin or Keppra versus those taking no medications (rows 5 and 7 of Table 1, we found the most common combination of hyper-parameters across cross-validation results and grid search to be: *C* = 10 or 100, $\gamma =0.1$, using 210 or 390 features found with K-best dimensionality reduction. Across all cross validations performed for each classification task (10-fold cross validation $\times$ six classifications = 60) when using kSVM models, the most common optimal hyper-parameters and dimensionality found through grid search were: *C* = 10, 100 and 1000; $\gamma = 0.1$; and 120, 210, or 390 features with PCA (all 570 features were found to be optimal in only 4 out of 60 cross validation runs). The most common hyper-parameters and dimensionality of linear SVM models were: *C* = 1 or 10; and 210 or 390 features with PCA.

Finally, comparing the computational time of training each classification method, we see in Table 2 that RFs classifiers were the fastest among all methods. Further, we see that there was little difference between SVM and kSVM models. For deep learning models we found that LNN, and SCNN, and EEGNet took nearly half the computation time than DCNN models. The implications of these classification, discriminability, and efficiency results are discussed in the Discussion section.

**Table 2:** Mean computation time in minutes (min) for each of seven classification methods.

Classification method	Feature-based (CPU)			Network-based (GPU)			
	SVM	kSVM	RF	LNN	SCNN	DCNN	EEGNet
Mean train time (min)	23.67 (0.20)	23.56 (0.20)	0.93	8.12	8.43	16.95	8.84

SVM and kSVM times reported for: mean train time with grid search; and in parentheses: mean train time with hyper-parameters and dimensionality reduction set. CPU: $30 \times$ Intel Xeon CPU E5-2630 v4 at 2.20GHz; GPU: 1 $\times$ NVIDIA GeForce GTX 1080 Ti (3584 CUDA cores with 11 Gbps memory speed).

## Discussion

The goals of the current study were to (1) determine if machine learning could predict medication state solely from EEG and (2) compare the accuracies of feature-based and deep learning classification methods. A very limited number of previous studies have looked at neurological marker differences using EEG between different anticonvulsant medications in order to assess the impact of the drugs on cognitive performance and neurological patterns^7^. To our knowledge, at the time of this publication, this study presents the first aiming to distinguish different anticonvulsant medications taken by patients using solely neurological activity though machine learning methods.

We first discuss the ability of advanced machine learning algorithms to classify the medication taken using only neurophysiological activity data. To better understand the clinical implications, we compare results using abnormal EEG which were the most significant classification results. Though there were no significant differences in classification accuracies between subjects taking Dilantin versus subjects taking no medications and subjects Keppra versus subjects taking no medications, there were significant differences in accuracies comparing each of the anticonvulsants versus subjects taking no medications and the classification results of subjects taking Dilantin versus subjects taking Keppra (Fig. 1a). Our comparisons of the cross-validated results imply that the clinical effects of Keppra and Dilantin on neural activity on subjects with abnormal EEG produced similar levels of discriminability to those not taking any medications. This is not an unexpected result given that both of these drugs are from the same class, anticonvulsant. Dilantin’s (phenytoin) mechanism of action, like most anti-epileptic drugs, is believed to act by limiting bursts of neuronal activity via blockade of sodium channels that give rise to the action potential^21^. However, Keppra’s (leviracetam) mechanism of action is less understood but believed to act through binding to the synaptic vesicle protein SV2A, resulting in reduced vesicle release^22^. Since anti-convulsants would be prescribed with the intent of reducing seizure activity, and since seizure activity has visible macroeffects in the EEG, it is not unexpected that they might show similar levels of discriminability. Though discriminability of each of the anticonvulsants versus subjects taking no medications were similar, further investigation would be needed to determine if the same differences were found for both medications.

Since the two medications used in this study are anticonvulsant drugs, without controlling for the clinical impression of the EEG, classifications found may have been based on the EEG pathology state rather than the differences in medications. By performing classifications on each group of subjects separately, we ensured that results found were not based on the presence of visible EEG abnormalities. Comparing the abnormal and normal EEG recordings, the abnormal EEG populations tests outperformed the normal EEG populations for most comparisons, except a few cases classifying between medications (rows 1 and 2 of Table 1). This result is logical considering the medications investigated are intended to treat those with abnormal EEGs and the expectation is that these drugs would have a more prominent effect on those with abnormal EEGs recorded. Furthermore, features used were drawn from EEG studies and have they have generally been designed to detect abnormalities in EEG which may have further added to this difference. While in the case of diagnosis, having the highest classification accuracy may be desirable, for medication classification, a better understanding of brain rhythms and neurological characteristics that separate the two populations may lead to better targeted drug therapies. The classification accuracies obtained in this study, all at or below 73%, do not achieve levels usually found in diagnostic applications^23^. However, they do provide promising results as a potential avenue for inexpensive, non-invasive, and more efficient methods to determine different neurological effects from drugs. Recently several large pharmaceutical manufactures have obtained approval to market existing medications for new uses using real-world data by analyzing medical records of subjects’ who had taken those medications^24,25^. These type of retrospective analyses combined with methods used in this study have the potential to accelerate the evaluation of safety and effectiveness of medications drastically by avoiding sometimes lengthy drug trials. Furthermore, several factors suggest that the present results may represent a lower bound on future classification performance with data of this type. These factors include continual increases in EEG resolution, electrode technology, and denoising techniques since the time that many of the TUH recordings were made, as well an expected manifold increase in the data available.

We discuss next the different machine learning algorithms and the advantages and disadvantages of each for this application. In the current study we obtain novel classifications for patient populations in the TUH data set based on medications taken. Recent works have begun to focus on comparing different machine learning methods in pharmaceutical research but there are still gaps in this area^18^. This work addresses one such gap, namely the relative performance of feature-based and deep learning model approaches. When compared to classification accuracies with randomly assigned medication status labels, we found significant results ($P<.01$) for deep learning classifications comparing subjects taking either one of the anticonvulsants against subjects taking no medications. We found that the DCNN models outperformed the LNN and EEGNet models in all cases and the SCNN models for all but one classification. However, the DCNN models were found to need twice as much computing time. Given these results, lightweight networks like the LNN and EEGNet were not able to learn underlying features needed to classify EEGs based on medications above chance. For our feature-based classifications we found significant classifications ($P<.01$) for all classifications. kSVM models consistently outperformed SVM model results with equivalent computing time. RFs models on the other-hand outperformed kSVM models except in two cases where results were not significantly different between kSVM and RF models. RF models also were the most computationally efficient to train among all methods. Using results from this work, if the hyper-parameters and dimensionality reduction methods for kSVM models were set and no grid search performed, then training would be more computationally efficient than RF models.

In general, feature-based classification accuracies were comparable to the deep learning classification accuracies and only RF models were found to be significantly different than SVM models in one classification (Fig. 1b). However, the standard deviations of the feature-based models were lower than the corresponding deep learning model results. This was expected given the stochastic nature of the optimization of DCNN models that produce larger variation in the cross-validated results in contrast to kSVM’s more deterministic approach. We also found that kSVM models yielded lower *P*-values than corresponding DCNN models even with lower cross-validated classification accuracies comparing subjects with abnormal EEG taking Dilantin versus those taking Keppra. Therefore, we see that the DCNN models were able to obtain accuracy percentages using random labels closer to results obtained from correctly labeled data. Given the automated nature of DCNN feature extraction and recently proven efficacy in complex data sets, DCNN models are expected to be able to find patterns where labels are non-informative^15^.

Further, since feature-based methods and deep learning methods utilize different computing resources, CPUs and GPUs, respectively, it is difficult to compare the computational efficiency of the two groups beyond the training time needed for these experiments. As expected, feature-based methods were less time-intensive than deep learning methods as long as the hyper-parameters were set. However, as the number of combinations in the grid search for optimal hyper-parameters increased, deep learning models were able to obtain similar performance in less time.

With all these factors, we find that kSVM models perform well from both an accuracy and efficiency perspective for medication status classification solely from EEG if there is domain expert knowledge available to determine application-specific features and hyper-parameters. If a more efficient method is desired, RF models would be an appropriate choice. When application-specific features are not available, DCNN models, though computationally intensive, provide better results. Though in this work we focus on feature-based approaches and feed-forward network models, future work may investigate different classification and deep learning models, such as recurrent neural networks and in particular long short-term memory (LSTM) networks which have been shown to perform well on sequential data like EEG, to improve medication classification from EEGs^4^. Further, as we found some models to perform better for certain classifications, future work may investigate different sets of medications to validate whether these models are still optimal for those types of classifications.

Finally, to understand the clinical relevance of these classifications and medication effects on neural activity, investigation into what characteristics of EEG are important to these classifications would be informative. There are several well established methods available to identify important characteristics in feature-based classifications^26^. Some of these methods were applied in this study along with cross-validation to identify subsets of features that performed best in SVM and kSVM models. Further, there are also established methods to identify feature importance in RF models. Future work may investigate which specific features were chosen in these classifications and their clinical implications. However, with respect deep learning classifications, the nature of these models limit our understanding of the important features in EEG when making classifications since the thousands, sometimes millions, of the features identified in the deep learning architecture remain a “black box”. This is in stark contrast to the discrete number of known features used in feature-based algorithms. Methods to investigate deep learning models have mainly been developed in the image processing space but have rarely been used in EEG^27^. However, recently, neurological differences between sexes and EEG pathologies have been investigated by attempting to understand what deep learning models distinguished using subjects’ EEGs^28,29^. Expanding available methods of investigating deep learning models using EEG would benefit understanding the underlying neurophysiological differences. Further research in explainable artificial intelligence to classifications investigated in this work, two anti-epileptic drugs with similar target patient populations, may better inform pharmacodynamic models.

## Methods

**Figure 2:**
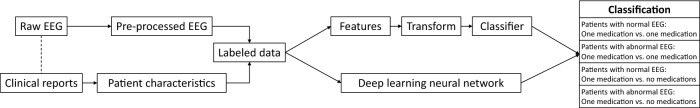
Data processing and classification flowchart. Labeled data, EEG signals, were classified through feature-based methods (top track) or deep learning-based methods (bottom track).

To investigate the different medication-based classifications, we applied several processing steps to EEG data obtained and corresponding clinical reports from the TUH EEG Corpus, version 1.0.0 (Fig. 2). This data set contains 23,257 EEG recording sessions (1.1 TB) from 13,551 subjects with a total of 61,802 recording files. Clinical EEG data were collected from records at Temple University Hospital (TUH)^9^ in accordance with human data guidelines and regulations. All data collection by TUH was performed in accordance with the Declaration of Helsinki and with the full approval of the Temple University institutional review board (IRB). All personnel in contact with privileged patient information were fully trained on patient privacy and were certified by the Temple IRB. We had no role in data collection or curation of the clinical database. Rather, data used in this study was obtained de-identified from the publicly available resource for secondary analyses.

Each session was accompanied by a report containing clinical impressions and patient characteristics, including age and sex, as well as the medications being taken by the patient at the time of recording^9^. The pre-processing methods, classification definitions, and machine learning approaches are described below. All pre-processing and analyses were done in Python using a combination of available packages^30^, published code from previous works^29^, and custom developed code (available at https://github.com/dbp-osel/medication-EEG-ML).

### EEG data

Since the recordings in the TUH database were not all acquired with the same EEG system, the channel layouts were not all consistent. A subset of channels common across most recording sessions was identified and used in subsequent analyses. From a standard 10–20 electrode montage, the subset of channels used were: Fp1, Fp2, F3, F4, C3, C4, P3, P4, O1, O2, F7, F8, T3, T4, T5, T6, Fz, Cz, Pz. For our deep learning models, the 19 input EEG channels were ordered in this manner. Any recording that did not include all of these channels was omitted from analysis.

Recordings without at least one recording 6 min in length were omitted from analysis. The first minute of each recording was excluded to reduce artifacts present in the EEG^29^. Our analyses were applied to the next five minutes of EEG recordings across those 19 channels identified above^31^. If another sampling rate was used, recordings were down-sampled to 100 Hz. The data were then filtered through a 0.5–50 Hz band pass filter, and re-referenced using a common average.

### Classifications: defining subject populations

A subset of patient clinical reports within the TUH data set had been previously reviewed and labeled by curators of this database to be clinically normal or abnormal^31^. The definitions of normal and abnormal used by the TUH generally followed methods neurologists use to identify abnormalities^32^. These labels were applied broadly to recordings where the clinical reports identified an abnormality in the EEG and do not distinguish between different abnormalities. An automated labeling method previously developed and validated based on textual analysis of the clinical reports was applied to categorize all available recordings as normal or abnormal^33^. Applying this method to the entire TUH data set resulted in a total of 6,001 EEG recordings with normal EEG across 4,058 unique subjects and 15,347 EEG recordings with abnormal EEG across 7,432 unique subjects.

Given the majority of clinical recordings from this data set were focused on diagnosing or monitoring individuals with epilepsy or seizure disorders, our medication classification focused on anticonvulsants. An initial textual analysis of the clinical notes for each EEG recording revealed hundreds of individuals taking the drugs Dilantin (generic name phenytoin) or Keppra (generic name levetiracetam), which are categorized as anticonvulsants^34^. For the purposes of this study, the clinical notes were used to determine which subjects were taking either Dilantin or Keppra, and which subjects were taking no medications. Any subject with more than one medication listed in the clinical notes was excluded from the evaluation to reduce results related to drug interaction effects. Furthermore, given the clinical nature of the data set and to avoid confounds from normal and abnormal EEGs, these classifications were performed separately on subjects with normal EEG and abnormal EEG.

Overall, six (three medication states $\times$ two EEG pathology states) different classifications were defined and analyzed using these data with seven different machine learning approaches. The population demographics of the entire data set used and each group of subjects are shown in Table 3.

**Table 3:** Population demographics across all medications of interest and no medications.

	All recordings	Taking Dilantin	Taking Keppra	No medications
Total *n*	35,370	718	898	5,1815
Normal EEG	6,001	179	175	1,203
Abnormal EEG	15,347	320	264	1,285
EEG status not obtained	14,022	291	459	2,693
Male	15,106	344	378	2,017
Female	16,434	320	465	1,895
Sex not obtained	3,830	54	55	1,269
Age (Mean ± SD years) (# recordings w/ age)	50.23 ± 19.15 ($n=33,021$)	41.14 ± 18.38 ($n=692$)	48.04 ± 18.98 ($n=862$)	44.55 ± 20.11 ($n=4,142$)
Unique subjects	13,486	456	507	2,530

Pre-processed data were linked to medication labels extracted from their corresponding clinical reports for seven machine learning approaches, described below. We note that other patient demographics (age and sex) were well balanced across all populations investigated.

### qEEG feature-based classifiers

#### qEEG features

From the pre-processed data, a set of time and frequency domain features were computed for each of the 19 channels on each recording. qEEG features used in this study were found in EEG literature and previously analyzed for inter- and intra-subject consistency^33,35,36^. Time domain features were computed on each five-minute recording. For spectral features, the Fourier transforms were taken on the five-minute pre-processed recording after which various spectral features were computed.

The power spectral density (PSD) of frequency bands commonly analyzed in the EEG were estimated using the periodogram. The ranges of the frequency bands applied in this study were as follows: $\delta \text{(delta) }$: 1–4 Hz, $\theta \text{(theta) }$: 4–8 Hz, $\alpha \text{(alpha) }$: 8–12 Hz, $\mu \text{(mu) }$: 12–16 Hz, $\beta \text{(beta) }$: 16–25 Hz, $\gamma \text{(gamma) }$: 25–40 Hz^8^. Both absolute powers and relative powers were computed, with relative power equal to the power in a frequency band divided by the total power. The entropy of the periodogram, and entropy of the normalized periodogram, were found using the Shanon entropy definition^37^. In addition to the spectral features, the following time domain features, directly from the pre-processed EEG signal, were computed: entropy of the normalized signal, mean thresholded Lempel-Ziv complexity (LZC), curve length, energy, non-linear energy, sixth power, minimum value, maximum value, median, variance, standard deviation, skew, kurtosis, integral, sum, mobility, complexity. The frequency and time domain features selected resulted in a total of 570 features per recording (30 features $\times$ 19 channels).

#### Feature transformation

Features were transformed using max normalization^38,39^. The max normalization transform was applied to the data by finding the mean and maximum value of each feature across all subjects in the training data. Each value, in both the training and testing data set, was then subtracted by the mean and divided by the maximum values obtained from the corresponding training set. This transformation scaled each feature to a range of values between zero and one in the training data and approximately between zero and one in the testing data set.

#### Support vector machine classifiers

A grid search was used to determine optimal dimensionality reduction method, and hyper-parameters for the linear SVM and non-linear kSVM classifiers. For the linear SVM classifiers and kSVM classifiers using a radial basis function (RBF) kernel, a grid search on 120 different combinations across: (1) dimentionality reduction methods $=${PCA, K-best}, (2) number of features used $=\{60, 120, 210, 390, 570\}$, of the available 570 features, (3) the penalty parameter $C = \{1, 10, 100, 1000\}$, and (4) the RBF smoothness parameter $\gamma =$ {0.1, 0.01, 0.001}, was performed for each classification for each model^30^. These training and grid searches were done on another 10-fold cross validation to tune the SVM and kSVM models using a validation set extracted from the initial training data set, independent from the testing set.

#### Random forest classifier

All computed and transformed features were used in a random forest classifier with 100 estimators (trees), two minimum samples to split a node (leaf), one minimum sample per node, no set maximum depth, and Gini impurity criterion^30^. A grid search on hyper-parameters was not performed for this method since we observed high training accuracies on all classifications as well as significant validation accuracies with these commonly used hyper-parameters.

### Deep learning neural network classifiers

**Figure 3:**
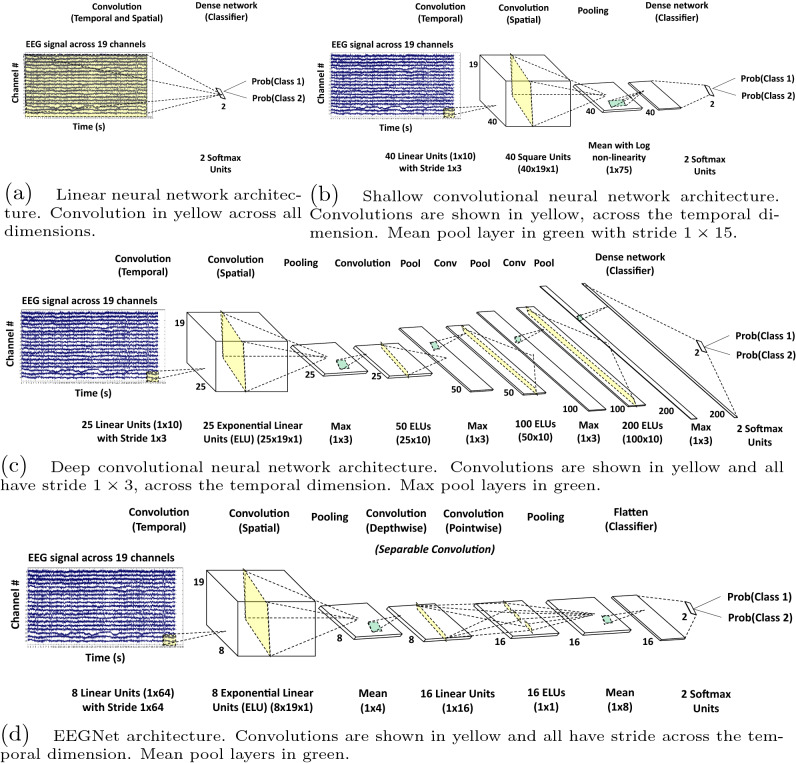
Deep learning neural network architectures: (**a**) linear neural network (LNN), (**b**) shallow convolutional neural network (SCNN), (**c**) deep convolutional neural network (DCNN), and (**d**) EEGNet. Classifications from each model was determined by the class with the highest output probability.

We used variants of a four deep learning models introduced in previous research shown in Fig. 3, some of which were applied to the abnormal TUH data set^29,31,40^. We mainly used feed-forward CNNs in this study because they have been the most common deep learning architecture successfully used in previous EEG research^4^.

All four models had weights randomly initialized. They were trained for 35 epochs for a first run and then restarted for a second run based on the epoch model with the highest training accuracy among the epochs from the first run. The second run’s stopping condition was based on a specified training loss to be met based on the first run’ results. These neural networks were optimized though the stochastic gradient descent inspired Adam algorithm^41^. A validation set was taken from 10% of the training set, independent of the testing data, for 10-fold cross-validation for model tuning. The application was run on an NVIDIA GTX-1080Ti for training and subsequent testing through Python 3.5 and Torch^29,42^.

#### Linear neural network

The first model we used was a simple LNN (Fig. 3a) which took as input EEG signals and applied a dense network layer with softmax activation functions to obtain prediction probabilities for each class. The class with the highest probability was the class determined by the deep network. This LNN model only required the number of weights equivalent to twice the size of the input EEG to be trained.

#### Shallow convolutional neural network

The second model we used was a SCNN (Fig. 3b) which also took as input EEG signals and applied two layers of convolutions and a pooling layer before a dense network layer for classification. The network applied batch normalization and dropout of 0.5 for the convolution and pooling pair^43,44^. The two-step convolutional sequence in the first two convolution layers was inspired in part by the filter-bank common spatial pattern (FBCSP) algorithm^40,45,46^. After the two convolutions, a mean pooling layer with logarithmic activation function together were analogous to the log-variance step in the FBCSP algorithm. The final layer applied a dense neural network with softmax activation functions to obtain prediction probabilities for each class. The class with the highest probability was the class determined by the deep network. This SCNN model required 38, 162 weights to be trained.

#### Deep convolutional neural network

The third model we used was a DCNN (Fig. 3c) which again took EEG signals as input and subsequently applied several layers of convolutions and pooling before a dense network classification layer. The main non-linearity used in many of the layers of this model was the exponential linear unit (ELU) function^47^. ELU is a strong alternative to the commonly used rectified linear unit (ReLU) function since it can generate negative outputs and decreases smoothly and slowly until its output equals the parameter value. This network also applied batch normalization and dropout of 0.5 with each convolution and pooling pair. The architecture used was similar to ResNet DCNN, which has been shown to be an effective neural network architecture for automated feature extraction^48^. Again, the first convolution and pooling pair was different from those used in most deep learning models because of the structure of EEG data. The two-step convolutional sequence in the first two convolution and pooling pairs was similarly inspired in part by the FBCSP algorithm. Since EEG data is a collection of one-dimensional signals rather than a two-dimensional array where the dimensions are coupled, such as an an image, the first convolution was applied to each channel separately, across time. The subsequent convolution then uses this result to apply a convolution on the resulting feature maps and channel collection, over time. Afterwards, three additional convolution and pooling pairs were applied to the resulting feature-maps in a more traditional fashion. The final layer took the final feature-map and applies a dense neural network with softmax activation functions to obtain prediction probabilities for each class. The class with the highest probability was the class determined by the deep network. This DCNN model required 279, 927 weights to be trained.

#### EEGNet

The fourth model we used was EEGNet (Fig. 3d) which took as input EEG signals and applied several layers of convolutions and pooling before flattening the output for classification with softmax activation functions to obtain prediction probabilities for each class. The ELU activation function is also used in this network as in the DCNN. Further, the first set of convolutions follow again the FBCSP inspired two-step sequence as shown before. However, the following set of convolutions applied are separable convolutions, done first depthwise, to summarize individual feature maps in time and then pointwise, to optimally combine the feature maps. This network applied batch normalization and dropout of 0.25 with each convolution and pooling pair. The final layer applies softmax directly to the flattened features to reduce the number of free parameters in the model. The class with the highest probability was the class determined by the deep network. This EEGNet model required 13, 296 weights to be trained.

### Classification results and significance

For each binary classification, an equal number of subjects from each population was used to ensure equal prevalence of each condition. 90% and 10% of the data were assigned randomly to a training and testing set, respectively. This process was repeated ten times to obtain classification test results using different subsets of the data for a 10-fold cross-validated set of results. To measure the significance of the results, we randomly assigned medication-status labels to the training data and again obtained ten cross-validated test set classifications based on random group labels. We compared the test set accuracy results of the ten models using correct labels and ten models using randomly labeled training data with a non-parametric Kruskal–Wallis test to determine the labels’ significance^49^. We used thresholds of $P< .01$ and $P<.001$ as two levels of significance. If the correct label test results were similar to the random label results and non-significant *P*-values were found, the labels were considered to be non-informative in the classification task.
